# A Novel Nonsense *GLI3* Variant Is Associated With Polydactyly and Syndactyly in a Family by Blocking the Sonic Hedgehog Signaling Pathway

**DOI:** 10.3389/fgene.2020.542004

**Published:** 2020-11-10

**Authors:** Ying Xiang, Xiaoliang Li, Zhiyan Zhan, Jue Feng, Haiqing Cai, Yanxin Li, Qihua Fu, Yunlan Xu, Hong Jiang, Xiaoqing Zhang

**Affiliations:** ^1^Shanghai Children’s Medical Center, Shanghai Jiao Tong University School of Medicine, Pediatric Translational Medicine Institute, Shanghai, China; ^2^Department of Pediatric Orthopedic, Shanghai Children’s Medical Center, Shanghai Jiao Tong University School of Medicine, Shanghai, China; ^3^Medical Laboratory, Huangshi Maternity and Children’s Health Hospital, Huangshi, China

**Keywords:** polydactyly, syndactyly, variant, *GLI3*, *SHH*, massively parallel sequencing

## Abstract

Polydactyly and syndactyly are congenital limb malformations that may occur either as non-syndromic or syndromic forms. In the present study, massively parallel sequencing was performed on a proband in a four-generation family with polydactyly and syndactyly to identify disease-causing variant(s). A pathogenic variant c.739C > T (p.Gln247^∗^) in the glioma-associated oncogene family zinc finger 3 (*GLI3*) gene was identified and co-segregated with the affected members of the family. Firstly, we examined *GLI3* mRNA and GLI3 protein levels in peripheral blood mononuclear cells (PBMCs) of patients carrying this variant. The results showed that the truncated GLI3 p.Gln247^∗^ (c.739C > T) protein was detectable in patients and the *GLI3* transcript and protein levels were not significantly altered in the PBMCs of patients compared with healthy controls. Furthermore, functional analysis showed that the truncated GLI3 p.Gln247^∗^ (c.739C > T) protein variant could lead to cytoplasmic accumulation of mutant protein and loss of ability to bind to the Suppressor of Fused protein. Alterations in protein expression levels of core components of the Sonic hedgehog signaling pathway were also observed. Our study shows that this novel *GLI3* variant contributes to the malformations in this family and provides evidence for the mechanism by which *GLI3* c.739C > T (p.Gln247^∗^) was implicated in the pathogenesis of polydactyly and syndactyly.

## Introduction

Glioma-associated oncogene homolog 3 (*GLI3*), a zinc finger transcription factor, is one of the most important downstream factors in the canonical Sonic hedgehog (SHH) signaling pathway. It acts as an essential regulator of limb development and tissue patterning (Vortkamp et al., 1991; Niedermaier et al., 2005; Biesecker, 2006; Hui and Angers, 2011; Al-Qattan et al., 2017; Matissek and Elsawa, 2020). In the SHH pathway, the membrane protein Patched 1 (PTCH1) is internalized with SHH, relieving inhibition of the accumulation of the signal transducer, Smoothened (SMO), on the ciliary membrane (Ahn and Joyner, 2004; Tickle and Towers, 2017). Suppressor of Fused (SUFU), a negative regulator of SHH signaling, can bind and maintain full-length GLI3 (GLI3FL) in the neutral state in the cytoplasm. In the absence of SHH signaling, the SUFU-GLI3FL complex is recruited to cilia, leading to efficient processing of GLI3FL into a repressor form (GLI3R) that suppresses SHH target gene expression in the nucleus. When SHH signaling is initiated, GLI3FL dissociates from SUFU and is transported to the nucleus, where it is phosphorylated, destabilized, and converted to the principal transcriptional activator of GLI3 (GLIA) (Humke and Rohatgi, 2010). Accumulation of GLIA to the nucleus enables activation of SHH target genes such as *PTCH1*, *GLI1*, and human hedgehog interacting protein 1.

The role of *GLI* genes in development was first revealed by the discovery of deleterious variants in *GLI3* in several human congenital malformations, including Pallister–Hall syndrome (PHS) (Kang et al., 1997), Greig cephalopolysyndactyly syndrome (GCPS) (Vortkamp et al., 1991), non-syndromic polydactyly (Radhakrishna et al., 1999), and acrocallosal syndrome (Elson et al., 2002). PHS is caused by the persistent formation of a mutant GLI3 protein with constitutive repressor function, and the clinical phenotype of the patients includes polydactyly, bifid epiglottis and/or hypothalamic hamartoma (Shin et al., 1999). GCPS, a dominant genetic disorder characterized by polydactyly and craniofacial features, is frequently associated with large deletions or truncating variants which result in loss of GLI3 function (Johnston et al., 2010; Abdullah et al., 2019).

In the present study, we investigated the genetic basis of polydactyly and syndactyly in a family with four members affected. Using massive parallel sequencing followed by Sanger sequencing, we identified a novel heterozygous non-sense variant c.739C > T (p.Gln247^∗^) in *GLI3* that segregated with the disease phenotype within the family. We found that the *GLI3* transcript and GLI3 protein levels were not significantly altered in the peripheral blood mononuclear cells (PBMCs) of patients compared with healthy controls. The truncated GLI3 p.Gln247^∗^ (c.739C > T) protein was detectable in the patients, but functional analysis showed that the GLI3 p.Gln247^∗^ variant led to cytoplasmic accumulation of mutant protein and loss of ability to bind to the Suppressor of Fused (SUFU) protein. Alterations in protein expression levels of core components of the SHH signaling pathway were also observed.

## Materials and Methods

### Case Report

Participants were examined at Renji Health Check-up Center and Child Healthcare Department at Shanghai Children’s Medical Center (Shanghai, China). Measurements were performed for the head shape, maxillofacial malformation, brain imaging, distance of the inner canthal, interpupillary distance, height, limb length, organa genitalia, anus, and throat (epiglottis). Anomalies were defined according to the recently published standard terminology (Biesecker et al., 2009; Hall et al., 2009). The study was approved by the ethics committee of Shanghai Children’s Medical Center, according to the Declaration of Helsinki, and written consent was obtained from all participants or legal guardians.

### Massively Parallel Sequencing and Criteria for Pathogenicity

Exome enrichment was performed using the TruSeq Exome Enrichment Kit and followed by 2 × 100 paired-end sequencing using a Hiseq 2000 Sequencing System (Illumina, United States). Sequence reads were produced by Illumina Consensus Assessment of Sequence and Variation v1.8 software, Illumina Off-Line Basecaller v1.8, and Illumina Sequencing Control v2.8. Sequence reads were aligned to the human reference genome using Burrows–Wheeler Aligner (Li and Durbin, 2010). Identification of variants was performed using Genome Analysis Tool Kit (GATK) and VarScan software (Koboldt et al., 2009; McKenna et al., 2010; Duitama et al., 2014). Single-nucleotide polymorphisms (SNPs) and insertions/deletions (indels) were filtered in different ways. All variants included in the most recent version of the National Center for Biotechnology Information dbSNP database were excluded. Low-frequency frame shift and truncating variants (minor allele frequency < 0.001) in any genes were considered potentially pathogenic. The pathogenicity of candidate variants was then assessed according to American College of Medical Genetics and Genomics (ACMG) standards and guidelines (Richards et al., 2015).

### Sanger Sequencing

The candidate variants identified above were validated by Sanger sequencing. The polymerase chain reaction (PCR) primers (forward: 5′-TGATGTGGGTTGTGTAAT GG-3′; reverse: 5′-AAGGGAACCAGAGCACCAG-3′) were designed using Primer 3^1^. For each fragment, PCR was conducted in a total reaction volume of 25 μL containing approximately 200 ng of genomic DNA, 12.5 μL of 2 × GC buffer I, 2.5 mmol/L MgCl_2_, 0.2 mmol/L dNTPs, 40 U/mL Taq DNA polymerase (Takara, Dalian, China), and 0.4 μmol/L of each primer. The PCR products were amplified for 35 cycles of the following: 95°C, 30 s; 60°C, 30 s; and 72°C, 50 s using an Eppendorf Mastercycler^®^ Pro thermal cycler (Eppendorf, Hamburg, Germany). PCR products were then subjected to DNA sequencing on an ABI 3130XL sequencer.

### RNA Extraction and Quantitative RT-PCR Assays

PBMCs were isolated from the whole blood of two affected patients (father and grandmother) and five healthy controls. Total RNAs were obtained using TRIzol (Invitrogen, Carlsbad, CA, United States) according to the manufacturer’s instructions, and cDNAs were synthesized from 1 μg of total RNAs using PrimeScript^TM^ RT reagent Kit (TaKaRa). *GLI3* mRNA levels in patients and controls were assessed using SYBR Premix Ex Taq (TaKaRa) and normalized to the housekeeping gene, *GAPDH*. Each experiment was conducted in triplicate and run on a CFX Connect Real-time PCR (BIO-RAD). Data evaluation was performed according to the 2^−ΔΔ*CT*^ method. The Student’s *t*-test was used for statistical comparisons, and differences were considered to be significant if the *P*-value < 0.05. Primers used for qRT-PCR are as follows:

*GLI3*-F 5′-TGGTTACATGGAGCCCCACTA-3′;
*GLI3*-R: 5′-GAATCGGAGATGGATCGTAATGG-3′;
*GAPDH*-F 5′-ACAACTTTGGTATCGTGGAAGG-3′;
*GAPDH*-R 5′-GCCATCACGCCACAGTTTC-3′.

### Plasmid Constructs

By removing Cas9 in lentiCas9-Blast (addgene#52962), we created a lenti-blast vector with new multiple cloning sites. Full-length wild-type *GLI3* cDNA tagged with Flag was cloned into the lenti-blast vector. *GLI3* variant vector was established using a Q5 Site-Directed Mutagenesis Kit (NEB, E0554S) following the manufacturer’s instructions. The target variant was validated by sequencing the mutated plasmid DNA clones in both sense and antisense directions, and any other variants were excluded.

### HEK293T Cell Transfection

HEK293T cells (obtained from the American Type Culture Collection) were cultured in Dulbecco’s Modified Eagle’s Medium high glucose containing 10% fetal bovine serum (Gibco, Life Technologies, Inc.) at 37°C in a humidified atmosphere containing 5% CO_2_. HEK293T cells were seeded in 10 cm dishes and were infected twice in the next 2 days at 24 h intervals. Viral supernatants were collected from HEK293T cells after transfection with wild-type or mutant *GLI3* lentiviral plasmids together with pMD2.G (Addgene# 12259) and psPAX2 (Addgene# 12260).

### Western Blotting Analysis

Nuclear and cytoplasmic proteins were extracted with Nuclear and Cytoplasmic Protein Extraction Kit and quantified with a BCA Protein Assay Kit (Beyotime Institute of Biotechnology, Beijing, China) according to the manufacturers’ instructions. PBMCs and total cell lysates were prepared in 1 × SDS buffer, directly analyzed by SDS-PAGE, and transferred onto nitrocellulose membranes. After blocking in 5% BSA in Tris-buffered saline containing 0.1% Tween-20, the membranes were incubated with antibodies against Flag (F18804, Sigma, Inc.), HA (51064-2-AP, Proteintech), GLI3 (ab6050, Abcam), GLI1 (ab49314, Abcam), PTCH1 (ab53715, Abcam), SHH (ab32281, Abcam), GAPDH (ET1702-66, Huabio, Hangzhou, China), and β-actin (ET1701-80, Huabio). The antigen–antibody complexes were incubated with HRP-conjugated secondary antibodies (Bioworld, Nanjing, China) and visualized by an infrared imaging technique (Bio-Rad Laboratories, Inc.). The intensity of protein bands was quantified using ImageJ^2^ to calculate the ratios of IntDen (GLI3)/IntDen (β-Actin) to ensure that the detection of protein bands was linearized. The Student’s *t*-test was used for the statistical comparison of GLI3 protein relative quantification between patients and normal controls.

### Immunoprecipitation of SUFU, GLI3, and GLI3 p.Gln247^∗^

HEK293T cells were seeded into 10 cm dishes 10 h prior to transfection with 3 μg of each of two expression constructs (HA-SUFU and Flag-GLI3 or Flag-GLI3 p.Gln247^∗^ and harvested 48 h after transfection. The cells were lysed with RIPA buffer (Beyotime Institute of Biotechnology, Beijing, China) for 30 min. The supernatants collected after centrifugation were incubated with Anti-FLAG^®^ M2 Magnetic Beads (Sigma, Inc.) for 16 h. Then, western blotting of the beads was performed as earlier described.

### Immunofluorescence

After seeding for approximately 24 h, HEK293T cells expressing the fusion proteins (Flag-GLI3 or Flag-GLI3 p.Gln247^∗^) were washed with phosphate-buffered saline (PBS) for at least three times and fixed with 4% paraformaldehyde in PBS for 15 min, permeabilized with 0.3% Triton X-100 in PBS for 15 min, and blocked with 10% goat serum in PBS for at least 2 h at room temperature. After washing twice with PBS, the cells were incubated with anti-Flag antibodies (1:500 diluted in PBS) overnight at 4°C and then incubated with secondary antibodies conjugated to Alexa 488 (1:500 diluted in PBS, Life Technologies). Nuclei were stained with DAPI, and then cells were observed under an immunofluorescence microscope (Carl Zeiss, Jena, Germany). Immunofluorescence images were analyzed by ImageJ software (see footnote) (Abraham et al., 2012). The ratio of fluorescence intensity data in the cytoplasm and nucleus was collected from three points at random in this two compartments of the cell separately, and the average value of the Flag fluorescence ratio was estimated from ten cells for the variant and wild type of GLI3 for getting mean ± standard deviation. Statistical differences were analyzed by *t*-test.

## Results

### Clinical Assessment of the Family With Polydactyly and Syndactyly

**FIGURE 1:**
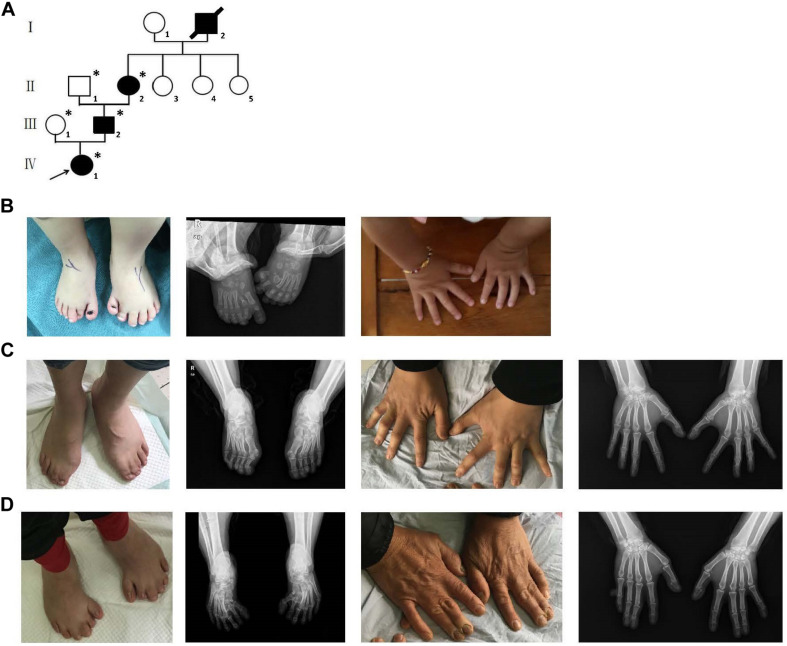
Clinical features of the affected members and family information. **(A)** Pedigree structure of a Chinese family with complex digital anomalies. Squares and circles denote males and females. Filled shapes indicate affected members. Individuals labeled with solidi are deceased. The arrow denotes the proband. Members marked with ^∗^ are the participants in this study. **(B)** Clinical features of the proband (IV-1) showing bilateral type I preaxial polydactyly of big toes with additional digits outside the great toe of the right foot and inside the great toe of the left foot. **(C)** Clinical features of the individual III-2 showing cutaneous syndactyly among the 3rd and 4th fused digits after the surgery and incomplete osseous syndactyly between the 1st and 2nd fused digits of both hands. Bilateral type I preaxial osseous polydactyly and syndactyly of big toes of both feet, and bilateral cutaneous syndactyly among the 1st, 2nd, and 3rd digits of both feet. **(D)** Clinical features of individual II-2 showing postaxial polydactyly in the right hand with cutaneous syndactyly between the 3rd and 4th digits of both hands, incomplete osseous syndactyly between the 1st and 2nd fused digits of the hands. Bilateral type I preaxial polydactyly of big toes with additional digits inside the great toes, and cutaneous syndactyly among the 2nd, 3rd, 4th, and 5th fused digits of both feet.

A four-generation family with polydactyly and syndactyly from Shanghai, China, was recruited to the study. No history of consanguineous marriage in this family was recorded according to the senior family members’ statements. The proband was a 5 year-old girl from Shanghai, China. Her head was symmetrical, with a normal circumference of 45.1 cm (reference range: 42.6–47.8 cm). Family history of the disorder was consistent with an autosomal dominant pattern of inheritance. In addition, three out of four affected individuals were examined in our study (Figure 1). Phenotypic variability was observed among affected individuals. The proband (IV-1) showed bilateral type I preaxial polydactyly of big toes, with additional digits outside the great toe of the right foot and inside the great toe of the left foot. Individual III-2 exhibited bilateral type I preaxial polydactyly of the big toes, with additional digits outside the great toes of both feet, and bilateral cutaneous syndactyly among the 1st, 2nd, 3rd, and 4th digits of both feet. Individual II-2 showed postaxial polydactyly in the right hand with cutaneous syndactyly between the 3rd and 4th digits of both hands, osseous syndactyly between the 1st and 2nd fused digits in the hands, and bilateral type I preaxial polydactyly of the big toes with additional digits inside the great toes of both feet (Figures 1B–D and Table 1). All of the affected individuals had a symmetrical head with normal circumference (reference range for males: 56.1–59.2 cm; reference range for females: 54.0–56.9 cm). No abnormalities of the shape of the head, maxillofacial malformation, brain imaging, distance of the inner canthal, interpupillary distance, height or limb length, organa genitalia, anus, and throat (epiglottis) was observed (Table 1).

**TABLE 1:** The symptoms of digit abnormalities in four affected members.

Individual	Gender	Age	Clinical feature	Finger	Toe
I1	Male	75 years	Hand bilateral Foot bilateral	Cutaneous syndactyly among the 3rd and 4th fused digits in both hands	Bilateral type I preaxial polydactyly of the big toes in both feet
II2	Female	50 years	Hand bilateral Foot bilateral	Postaxial polydactyly in the right hand with cutaneous syndactyly between the 3rd and 4th digits in both hands, and osseous syndactyly between the 1st and 2nd fused digits in both hands	Bilateral type I preaxial polydactyly of big toes with additional digits inside the great toes in both feet
III2	Male	28 years	The left hand Foot bilateral	Cutaneous syndactyly among the 3rd and 4th fused digits in the hands after surgery. and osseous syndactyly between the 1st and 2nd fused digits in both hands	Bilateral type I preaxial polydactyly of big toes with additional digits outside the great toes in both feet, and bilateral cutaneous syndactyly among 1st, 2nd, 3rd, and 4th digit in both feet
IV 1	Female	5 years	Foot bilateral	/	Bilateral type I preaxial polydactyly of big toes with additional digits outside the great toe in the right foot and inside the great toe in the left foot

### Variants Identified by Massively Parallel Sequencing

**FIGURE 2:**
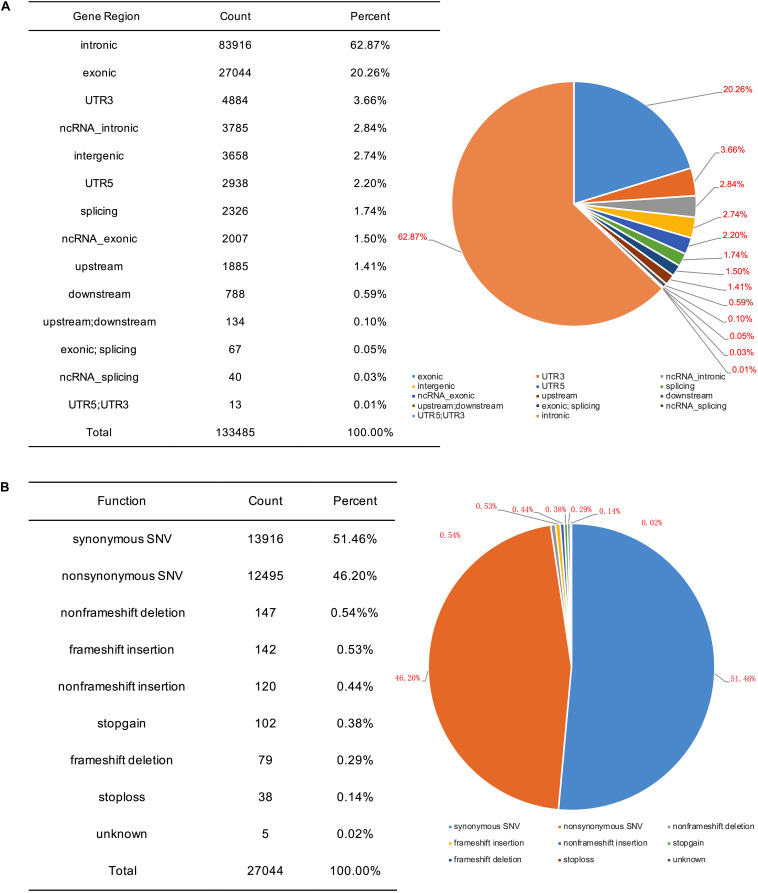
SNV information obtained by massively parallel sequencing. **(A)** Genomic distribution of SNVs. **(B)** Functional category of SNVs.

To identify potential pathogenic gene variants in this family, massively parallel sequencing was performed on the proband. Approximately 72.05M reads were obtained, of which 96.87% of the reads had a Q score of > 30 (Table 2). About 133,443 single-nucleotide variations (SNVs) were obtained, and 27,044 SNVs (20.26%) were located within exonic regions (Figure 2A). Detected variants included frameshift deletions, frameshift insertions, non-frameshift deletions, non-frameshift insertions, non-synonymous SNVs, stop gain SNVs, and synonymous SNVs (Figure 2B). After bioinformatic analysis, a novel non-sense variant, *GLI3* c.739C > T (p.Gln247^∗^), was identified as the candidate disease-causing variant in the proband (Table 3).

**TABLE 2:** Summary of WES data.

	IV-1
Reads Num(M)	72,049,850
Base(G)	10,611,136,501
ON BAIT BASES(M)	6,250,861,508
PCT_TARGET_BASES_2X	98.79%
PCT_TARGET_BASES_10X	95.35%
PCT_TARGET_BASES_20X	87.91%
PCT_TARGET_BASES_30X	78.50%
Q20	98.58%
Q30	96.87%

**TABLE 3:** The gene list after filtering based on phenotype using ingenuity variant analysis.

CHR	Position	Variation type	Gene symbol	Transcript ID	Transcript variant	Protein variant	Genotype
3	14,561,629	Missense	HOXD11	NM_021192	c.734 G > A	p.G245D	Het
7	42,085,070	Stop gain	GLI3	NM_000168.5	c.739 C > T	p.Y247*	Het
22	20,779,975	Insertion	SCARF2	NM_153334.6	c.2292dupG	p.E765fs*9	Het
22	20,780,032	Insertion	SCARF2	NM_153334.6	c.2234dupC	p.R746fs*28	Het
X	15,286,4480	Insertion	FAM58A	NM_001130997.2	c.54dupG	p.Q19fs*39	Het
X	152,864,515	Insertion	FAM58A	NM_001130997.2	c.17dupG	p.G7fs*51	Het

### Validation of the *GLI3* c.739C > T (p.Gln247^∗^) Variant

**FIGURE 3:**
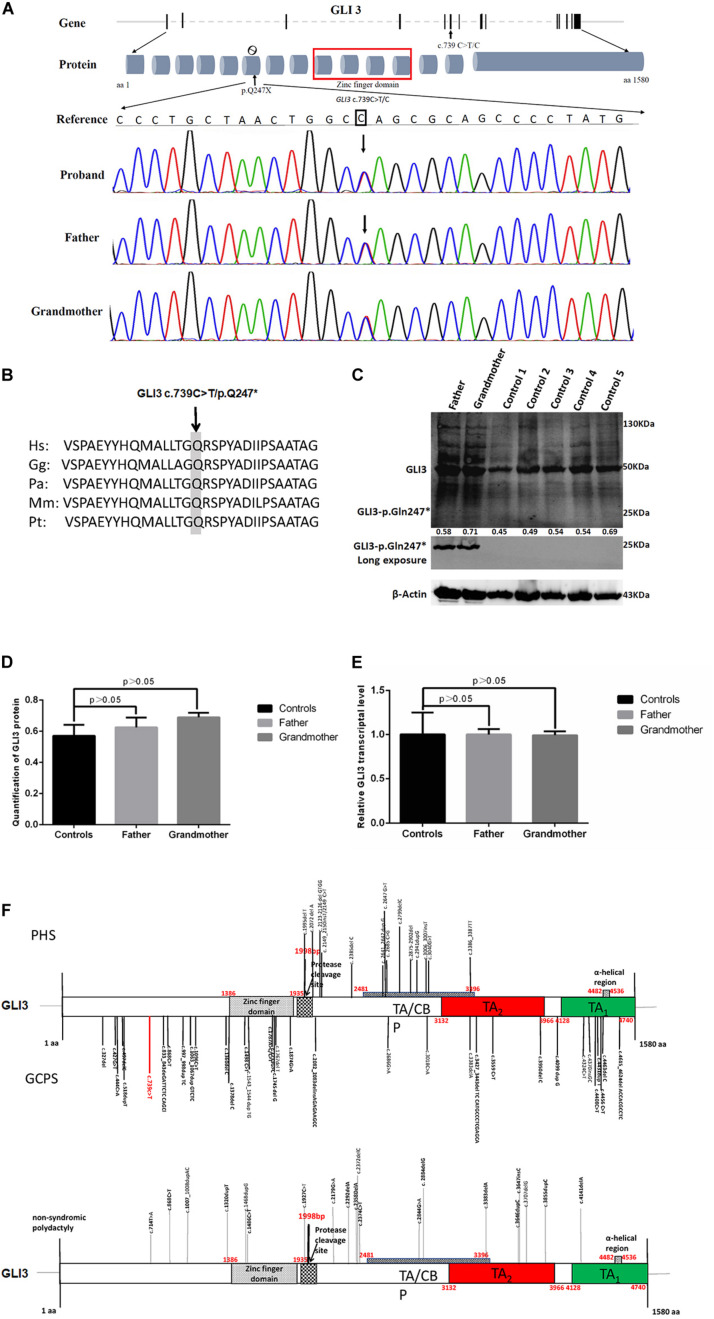
Novel *GLI3* c.739C > T variant in members of the polydactyly and syndactyly family. **(A)** The location of c.739C > T within GLI3 gene and Sanger sequencing results of the mutated region. From top to bottom: human reference sequence, followed by the PCR sequence of three affected members (IV-I, III-2, II-2) with the variant c.739 C > T and an unaffected member (III-1). **(B)** Comparison of partial amino acid sequence of human GLI3 with other primates. The shaded amino acid indicates the conserved residue across different primate species. Species abbreviations are as follows: Hs, Homo sapiens; Gg, Gorilla gorilla; Pa, Pongo abelii; Mm, Macaca mulatta; Pt, Pan troglodytes. The NCBI identifiers for the respective proteins are as follows: Hs, NP_000159.3; Gg, XP_0040-45386.1; Pa, XP_009241116.1; Mm, XP_001098108.1; Pt, NP_001029362.1. **(C)** Evaluation of GLI3 protein levels in PBMCs from patients with polydactyly and syndactyly and normal controls by western blotting. **(D)** The statistical comparison of GLI3 protein relative quantification showed that there was no significant difference between patients and normal controls (*p* > 0.05). **(E)** qRT-PCR analysis shows that the *GLI3* transcript is not significantly altered in PBMCs of patients compared with healthy controls (*p* > 0.05). **(F)** Distribution of PHS, GCPS, and non-syndromic polydactyly variants on the *GLI3* gene.

Family members were screened for the *GLI3* c.739C > T (p.Gln247^∗^) variant by Sanger sequencing. Sanger sequencing revealed that only the proband and other affected individuals in this family were heterozygous for *GLI3* c.739C > T (p.Gln247^∗^) (Figure 3A). Therefore, this variant co-segregated with the polydactyly and syndactyly phenotype in the family. Comparison of the amino acid sequences indicates that the Gln residue in codon 247 of GLI3 is conserved across different primate species (Figure 3B). The *GLI3* variant detected in this study is located upstream of the zinc finger domain (Figure 3F).

Based on the ACMG guidelines, the *GLI3* c.739C > T (p.Gln247^∗^) variant was classified as likely pathogenic based on the following criteria: PVS1_Very Strong, PM2_Moderate.

### Validation of Non-sense-Mediated mRNA Decay (NMD)

As the patients’ bone tissues were unavailable, we examined GLI3 mRNA and protein expression levels in PBMCs isolated from available peripheral blood samples of the two affected patients (father and grandmother). The results of RT-qPCR and western blotting showed that the GLI3 transcript and protein levels were not significantly altered in the PBMCs of patients compared with healthy controls (Figures 3C–E, *p* > 0.05). The truncated GLI3 p.Gln247^∗^ (c.739C > T) protein was detectable in the PBMCs of patients.

### Functional Studies on the GLI3 p.Gln247^∗^ Variant

**FIGURE 4:**
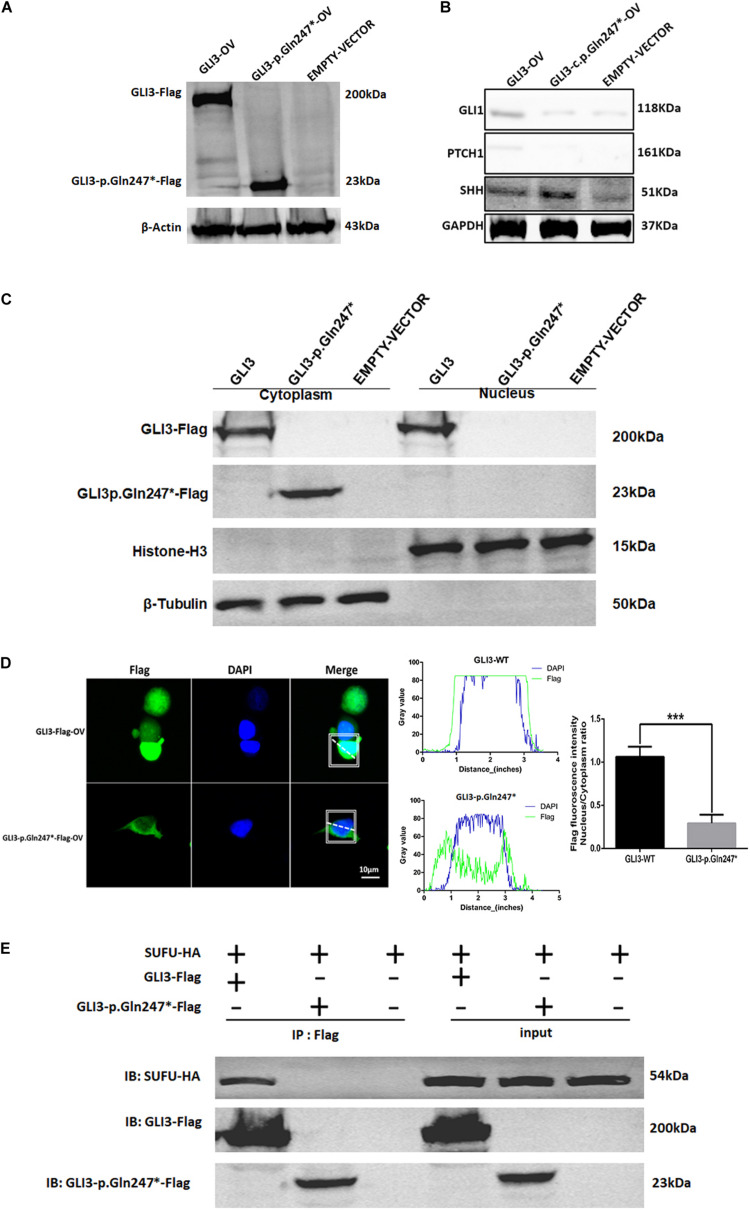
The novel GLI3 p.Gln247^∗^ variant blocks the SHH pathway. **(A)** Western blotting analysis of the ectopic expression of GLI3 wild-type and mutant plasmids in HEK293T cells. **(B)** Expression levels of core proteins in the SHH signaling pathway in wild-type and mutant GLI3 overexpression HEK293T cells. Expression of PTCH1 and GLI1 is lower in the HEK293T cells with the mutant GLI3 plasmid or empty vector than with GLI3FL. **(C)** Localization of GLI3-Flag and GLI3 p.Gln247^∗^-Flag in HEK293 cells by immunoprecipitation. **(D)** Localization of the wild-type GLI3 and p.Gln247^∗^ GLI3 protein in HEK293T cells. Nucleocytoplasmic separation detection of GLI3 wild-type and p.Gln247^∗^ protein in HEK293T cells exhibited cytoplasmic accumulation. Confocal microscopy showed that wild-type GLI3 protein localized within the cytoplasm and nucleus, and the GLI3 p.Gln247^∗^ variant protein was strongly detectable in cytoplasm but barely in the nucleus. To quantitatively compare the intracellular localization ratio of the GLI3 wild-type and p.Gln247^∗^ protein in the nucleus and cytoplasm, fluorescence intensity data was collected from three points at random in these two compartments of the each cell separately. The average nucleus/cytoplasm intensity ratio of flag fluorescence was calculated from 10 HEK293T cells expressing GLI3 wild-type and p.Gln247^∗^ variant protein separately. Statistical differences were analyzed by *t*-test. Histograms showed that the ratio of flag fluorescence distribution in the nucleus and the cytoplasm was significantly different between wild type and p.Gln247^∗^ transfected cells (*p* < 0.0001). **(E)** Immunoprecipitation and western blotting assays of the binding of SUFU with wild-type and mutant GLI3 protein. IB: immunoblotting.

To explore whether the GLI3 non-sense variant detected in our study affected the SHH signaling pathway, we constructed GLI3 wild-type and GLI3 p.Gln247^∗^ flag fusion protein lentivirus plasmids and transfected these into HEK293T cells separately. Western blotting analysis showed that the GLI3 p.Gln247^∗^ protein is shorter than the wild type (Figure 4A). Compared with the wild-type GLI3, the shortened GLI3 protein reduced the expression of PTCH1 and GLI1, whereas the expression of SHH was not disrupted (Figure 4B). Then, the wild-type and mutant plasmids were transfected into HEK293T cells separately to investigate whether p.Gln247^∗^ affected the intracellular location of GLI3. First, cytoplasmic and nuclear proteins of the transfected cells were isolated. Western blotting analysis showed that wild-type GLI3 was distributed in both the cytoplasm and nucleus, whereas the mutant GLI3 was strongly detectable in cytoplasm (Figure 4C). Immunostaining of GLI3, and of nucleus markers, was exploited to trace intracellular GLI3 trafficking through the Sonic hedgehog pathway. Confocal microscopy followed by analysis of the two fluorescence signals clearly localized wild-type GLI3 protein within cytoplasm and nucleus (Figure 4D). Conversely, the GLI3 p.Gln247^∗^ variant protein was strongly detectable in the cytoplasm but barely in the nucleus. Histograms showed that the ratio of GLI3 distribution in the nucleus and the cytoplasm was significantly different between wild-type and p.Gln247^∗^ transfected cells (*p* < 0.0001).

In SHH signaling, SUFU functions as an inhibitor by binding GLI3FL proteins in the cytoplasm and preventing them from reaching the nucleus. In our study, immunoprecipitation (IP) and western blotting assays showed that the GLI3 p.Gln247^∗^ variant protein could not bind to SUFU (Figure 4E), indicating that the truncated protein of GLI3 destroyed the SUFU-GLI3 regulatory axis.

## Discussion

GLI3 protein can be functionally divided into three parts: the N-terminal part, the middle part, and the C-terminal part (Al-Qattan et al., 2017; Figure 3F). Previous studies have shown that variants in different regions of GLI3 may lead to different congenital limb malformations such as PHS, GCPS, acrocallosal syndrome, and non-syndromic polydactyly (Démurger et al., 2015). For PHS and GCPS, a strong genotype–phenotype correlation with *GLI3* variants has been well described (Biesecker, 1997; Johnston et al., 2005; Krauβ et al., 2009; Johnston et al., 2010; Jamsheer et al., 2012; Abdullah et al., 2019). The N-terminal part of GLI3 contains the zinc finger domain (ZFD, aa462–aa645) and truncation variants upstream of or within ZFD, which may influence the functions of the activator or repressor forms of GLI3, usually result in GCPS. The middle part of the GLI3 protein contains the proteolytic cleavage site (PC, aa703–aa740) and truncation variants in this site, which result in extreme abundance of the GLI3R within the limb buds and neural tube, usually causing PHS (Al-Qattan et al., 2017). The C-terminal part of the GLI3 protein contains the transactivation domains 1 and 2 (TA_2_, aa1044–aa1322; and TA_1_, aa1376–aa1580). Variants in the C-terminal activator domain are predicted to cause the loss of transactivation domains (Shin et al., 1999) and may lead to variable phenotype including GCPS. It was also showed that GCPS can be caused by point variants in the C-terminal part of GLI3 which is related to the misregulation of its subcellular localization secondary to abnormal functional interaction (Krauβ et al., 2009).

Johnston et al. (2010) summarized a large cohort of patients with a wide phenotypic spectrum and demonstrated that pathogenic *GLI3* variants can cause malformations that are milder than the typical GCPS or PHS, and a relaxation of the clinical criteria for them would identify additional patients. Patients were categorized as being sub-GCPS when they presented one or more features of GCPS, including preaxial polydactyly, cutaneous syndactyly, widely spaced eyes, or macrocephaly, but did not meet the typical and clinically defined criteria for GCPS (Johnston et al., 2010). In our research, the major features of affected members in the family were preaxial polydactyly, broad thumbs or great toe, and syndactyly, which met the recommended clinical criteria of sub-GCPS. Meanwhile, we successfully identified the *GLI3* c.739C > T (p.Gln247^∗^) variant as the disease-causing variant for this four-generation family, which was located in the N-terminal of GLI3 protein (Figure 3F). Considering that truncation variants within N-terminal part are highly correlated with GCPS and sub-GCPS, we believed that the affected individuals can be classified into patient group of sub-GCPS.

Maintaining the appropriate balance between GLI3A and GLI3R is critical for tissue patterning during development (Al-Qattan et al., 2017). The dissociation of GLI3 from SUFU is pivotal for this balance. However, the mechanism by which this dissociation of GLI3 from SUFU was promoted remains unclear. Some insights into this key step come from the finding that activation of protein kinase A (PKA) can prevent the dissociation of the GLI3FL–SUFU complex, leaving GLI3FL stranded in the cytoplasm and making it unable to activate target genes (Chen et al., 2009; Krauβ et al., 2009).

In our study, the *GLI3* transcript and GLI3 protein levels were not significantly altered in the PBMCs of patients compared with healthy control (Figures 3C–E, *p* > 0.05). The truncated GLI3 p.Gln247^∗^ (c.739C > T) protein was detectable in the patients, and it can constitutively block the SHH signaling pathway, abrogate the function of GLI3, lead to the loss of binding ability with SUFU, restrain GLI3 from entry into the nucleus as a regulator, and influence the expression of key functional proteins of the SHH signaling pathway in a family with polydactyly and syndactyly.

## Conclusion

Our study shows that this novel *GLI3* variant (*GLI3* c.739C > T/p.Gln247^∗^) contributes to the malformations in a four-generation family with polydactyly and syndactyly and provides evidence for the mechanism by which the GLI3 p.Gln247^∗^ implicated in the pathogenesis of polydactyly and syndactyly.

## Data Availability Statement

The datasets generated for this study can be found in the PRJNA609774.

## Ethics Statement

The studies involving human participants were reviewed and approved by the Ethics Committee of Shanghai Children’s Medical Center. Written informed consent to participate in this study was provided by the participants’ legal guardian/next of kin. Written informed consent was obtained from the individual(s), and minor(s)’ legal guardian/next of kin, for the publication of any potentially identifiable images or data included in this article.

## Author Contributions

XZ, HJ, and YXu: conceptualization. XZ and YXi: formal analysis. YXi, XL, ZZ, JF, HC, YL, and QF: investigation. YXi: writing—original draft preparation. XZ, HJ, YXu, QH, and YL: writing—review and editing. All authors contributed to the article and approved the submitted version.

## Conflict of Interest

The authors declare that the research was conducted in the absence of any commercial or financial relationships that could be construed as a potential conflict of interest.

## Footnotes

1 http://bioinfo.ut.ee/primer3-0.4.0/primer3/

2 https://imagej.net
